# Judgment of Learning Accuracy in High-functioning Adolescents and Adults with Autism Spectrum Disorder

**DOI:** 10.1007/s10803-016-2895-1

**Published:** 2016-08-26

**Authors:** Catherine Grainger, David M. Williams, Sophie E. Lind

**Affiliations:** 1 School of Natural Sciences, University of Stirling, 3B101, Cottrell Building, Stirling, FK9 4LA UK; 2 School of Psychology, Keynes College, University of Kent, Canterbury, CT2 7NP UK; 3 Autism Research Group, Department of Psychology, City University London, Northampton Square, London, EC1V 0HB UK

**Keywords:** Autism, Memory, Metacognition, Metamemory, Judgment of learning, Theory of mind, Mindreading

## Abstract

This study explored whether adults and adolescents with autism spectrum disorder (ASD) demonstrate difficulties making metacognitive judgments, specifically judgments of learning. Across two experiments, the study examined whether individuals with ASD could accurately judge whether they had learnt a piece of information (in this case word pairs). In Experiment 1, adults with ASD demonstrated typical accuracy on a standard ‘cue-alone’ judgment of learning (JOL) task, compared to age- and IQ-matched neurotypical adults. Additionally, in Experiment 2, adolescents with ASD demonstrated typical accuracy on both a standard ‘cue-alone’ JOL task, and a ‘cue-target’ JOL task. These results suggest that JOL accuracy is unimpaired in ASD. These results have important implications for both theories of metacognition in ASD and educational practise.

## Introduction

Autism spectrum disorder (ASD) is a developmental disorder diagnosed on the basis of social-communication deficits, and fixated interests and repetitive behaviours (American Psychiatric Association 2013). There is substantial evidence that, at the cognitive level, individuals with ASD manifest impairments in representing *others’* mental states (“mindreading” or “theory of mind”; see Yirmiya et al. 1998) and that this contributes to social-communication features of ASD (see Brunsdon and Happé 2014). Recently, however, studies have begun to find evidence that individuals with ASD also manifest difficulties with representing their *own* mental states (so-called “metacognition”).

There is now reasonably consistent evidence that individuals with ASD manifest high rates of alexithymia, the inability to accurately identify and describe one’s own emotions (Hill et al. 2004; Silani et al. 2008), and show diminished performance in “self-versions” of classic mindreading tasks, in which they are required to explain their own behaviour in terms of underlying mental states (e.g., Williams and Happé 2009). However, very little is known about the extent to which individuals with ASD are able to monitor other aspects of cognitive activity in themselves. The ability to represent one’s own current, online mental states and cognitive activity is termed metacognitive monitoring, and is important for everyday self-regulation of behaviour and learning. For example, to study for an exam successfully, an individual needs to accurately monitor what information they already know and what they still need to learn. In this way, they can modify their learning/study behaviour accordingly, and spend more time studying appropriate information. Indeed, studies that have shown that several educational outcomes (such as exam performance) can be predicted by metacognitive monitoring accuracy, (e.g., Hartwig et al. 2012; Thiede et al. 2003). Given that individuals with ASD often demonstrate difficulties self-regulating their behaviours, and often underperform at school relative to IQ-matched peers (see Estes et al. 2011; Jones et al. 2009), the study of metacognitive monitoring in ASD is important.

Metacognitive monitoring is usually assessed by asking individuals to make online judgements about the current state of their knowledge or learning. For example, in “feeling of knowing” (FOK) tasks, participants are asked to predict whether they would be able to correctly recognise a memory target that they cannot currently recall (e.g., an individual might fail to recall the capital city of Australia, but still feel confident that they would recognise the correct city (Canberra) when presented with a list of several options). Alternatively, in standard judgement of confidence (JOC) tasks participants are typically asked to make retrospective judgements assessing how confident they are that their answer to a question is correct. The few existing studies of metacognitive monitoring in ASD have suggested that individuals with this disorder demonstrate diminished accuracy when making both feeling of knowing judgements (Grainger et al. 2014; Wojcik et al. 2013) and judgments of confidence (Grainger et al. 2016; Wilkinson et al. 2010; Brosnan et al. 2015; McMahon et al. 2016; but see Sawyer et al. 2014). That is, the correspondence between participants’ predictions about their own memory performance and their actual memory performance appears to be lower among individuals with ASD than among neurotypical individuals, when making these types of metacognitive judgments. However, one crucial type of metacognitive judgement that has barely been explored in ASD involves monitoring one’s own current state of *learning*.

During a standard judgement of learning (JOL) task participants are initially asked to memorise a series of stimulus pairs (e.g., pairs of words, such as “pen-key”, “computer-elephant” etc.). After this study phase, participants completed a JOL phase. During this phase, participants are sequentially presented with one stimulus from each pair (the cue; e.g., “pen”) and asked to make a judgement about the likelihood that, at a later point, they will be able to remember its accompanying, paired stimulus (the target; i.e., “key”). Finally, during a recall phase, participants are presented with each cue stimulus in turn and asked to recall the corresponding missing target stimulus. The accuracy of participants’ metacognitive judgements is measured by comparing participants’ judgments about their future recall performance with their actual recall performance.

To date, only one study has explored JOL accuracy in individuals with ASD (Wojcik et al. 2014). In one experiment (Experiment 1), adolescents with ASD and neurotypical adolescents were visually presented with word pairs during a study phase. Participants were then either asked to make immediate judgements (after each study trial) about whether they would be able to remember the target words (an immediate JOL task) or to make JOL decisions after a delay (during a delayed JOL task). The accuracy of participants’ judgments was assessed using Gamma correlations (Goodman and Kruskal 1954). Gamma correlations are the standard measure used to assess metacognitive accuracy on JOL tasks, and measure the association between individuals’ predictions about whether they have learnt a piece of information with their subsequent memory for that piece of information on a recall task (see the Method section for a more detailed description of how Gamma correlations are calculated). Wojcik et al. (2014) reported that adolescents with ASD were as accurate as neurotypical participants at judging their future memory performance, across both the immediate and delayed JOL tasks. Additionally, in a second experiment (Experiment 2), adolescents with ASD and neurotypical adolescents were asked to make delayed JOL decisions for both easy word pairs (made up of concrete nouns, e.g. paper-water) and hard word pairs (made up of abstract nouns, e.g. dream-fluency). Again, for both easy and difficult word pairs, Wojcik et al. (2014) found no group difference in JOL accuracy, and concluded that individuals with ASD were as good at making accurate judgements of learning as neurotypical individuals.

However, there are several reasons to be cautious when drawing conclusions from Wojcik et al.’s (2014) study. First, there was strong indication that monitoring accuracy was impaired in individuals with ASD in at least one respect. In the immediate JOL task (reported in Experiment 1), the mean gamma score was .05 (*SD* = .11) among participants with ASD and .27 (*SD* = .11) among comparison participants. Although the difference between groups in accuracy was not statistically significant, it was associated with a very large effect size, according to our calculations (Cohen *d* = 2.00). In other words, metacognitive monitoring in this condition did appear to be diminished in participants with ASD. Indeed, Wojcik et al. reported that performance was not even significantly above chance among participants with ASD, making an accurate interpretation of between-group differences difficult.

Secondly, in Wojcik et al.’s (2014) Experiment 2, there is some ambiguity about which participants were included in the analysis of gamma scores (i.e., JOL accuracy). Although their Table 3 (p. 401) reports gamma scores among *n* = 19 participants per diagnostic group, the ANOVA conducted with Gamma score as the dependent variable is associated with only 26 degrees of freedom (see Wojcik et al. 2014, pp. 401–402). In that case, it is unclear whether participant groups were matched for the relevant baseline characteristics (see Mervis and Klein-Tasman 2004, for a discussion of the importance of group matching). Given (a) these uncertainties about Wojcik et al.’s findings, (b) that Wojcik et al.’s findings are out of keeping with the findings of studies that have investigated other types of metacognitive judgement, and (c) the important role JOL accuracy plays in everyday learning, a further investigation of this issue among closely matched groups of ASD and comparison participants is timely.

The aim of the current study was to accurately assess JOL accuracy in individuals with ASD. The design we employed was similar to the design employed by Wojcik et al. (2014) in their delayed JOL condition, but among closely matched groups of participants so as to remove potential ambiguity in interpreting experimental results. Our main prediction for Experiment 1 was that adults with ASD would demonstrate diminished JOL accuracy, reflecting impaired metacognitive monitoring (in keeping with findings from studies of other types of metacognitive judgement).

## Experiment 1: Method

### Participants

Ethical approval for this study was obtained from Durham University Psychology Research Ethics Committee. Eighteen adults with ASD (13 males, 5 females) and 18 neurotypical comparison adults (11 males, 7 females) took part, all of whom gave written, informed consent before participating. Participants in the ASD group had all received formal diagnoses of autistic disorder (*n* = 4) or Asperger’s disorder (*n* = 14), according to DSM-IV or ICD-10 criteria (American Psychiatric Association 2013; World Heath Organisation 1993). In order to assess current ASD features, 15 of the 18 participants in the ASD group completed Autism Diagnostic Observation Schedule-Generic (ADOS; Lord et al. 2000) assessments (which were administered by a trained, research-reliable assessor). The ADOS is a semi-structured, standardized assessment of communication, social interaction, and imaginative use of materials that can be used to help diagnose ASD. The remaining three participants declined to complete the ADOS, as they did not feel comfortable being filmed. Each of the three participants who did not complete the ADOS had a confirmed ASD diagnosis and scored above the cut-off on the Autism-spectrum Quotient (AQ; Baron-Cohen et al. 2001), a self-report questionnaire that assesses ASD/ASD-like features. All participants who completed the ADOS received a total score ≥7, the defined cut-off for ASD (Lord et al. 2000). All participants completed the AQ questionnaire. Fifteen out of 18 participants with ASD scored above the defined cut-off for ASD on the AQ (total score ≥26; Woodbury-Smith et al. 2005). Only three participants missed this cut-off. However, all three of these participants scored well above the defined ASD cut-off on the ADOS (all ADOS scores among these three participants were ≥12). All comparison participants scored below the defined cut-off for ASD on the AQ.

No participants, in either group, reported using any psychotropic medication or any history of neurological or psychiatric disorders (apart from ASD). The participant groups were closely equated for verbal and non-verbal ability (see Table 1 for participant characteristics). Groups were also closely equated for chronological age and sex.

**Table 1:** Participant characteristics (means, standard deviations and inferential statistics) for the participants in Experiment 1

	Group		*t*	*p*	Cohen’s *d*
	ASD (*n* = 18)	Neurotypical (*n* = 18)			
Age (years)	28.96 (10.28)	30.43 (14.59)	0.35	.730	0.12
VIQ	111.67 (14.66)	112.28 (10.87)	0.14	.888	0.05
PIQ	109.67 (15.75)	114.50 (10.96)	1.07	.293	0.36
FSIQ	112.33 (15.00)	114.94 (10.50)	0.61	.549	0.20
AQ total score	33.39 (9.24)	13.00 (6.22)	7.77	<.001	2.59
ADOS social + communication score^a^	11.93 (2.19)				

*AQ* Autism-spectrum Quotient, *ADOS* Autism Diagnostic Observation Schedule, *PIQ* performance IQ, *FSIQ* full scale IQ, *VIQ* verbal IQ

^a^Based on 15/18 participants

### Materials and Procedures

#### Judgement of Learning Task

**Fig. 1:**
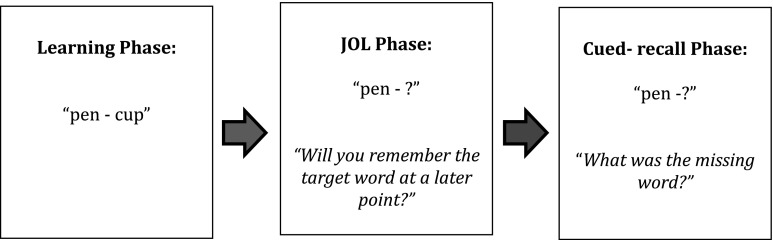
Graphical representation of the JOL task used in Experiment 1

A delayed JOL design was employed, consisting of a study phase, a JOL phase, and a cued-recall test phase (please see Fig. 1 for a graphical representation of the task). The stimuli used during the JOL task were 80 word pairs (160 words) all of which all were concrete nouns. Each word pair was made up of a “cue” word, which was used as a cue in both the JOL and cued-recall test phase, and a “target” word, which participants were not presented with during the JOL or cued-recall phase. Cue words and target words were matched for word frequency (Kucera and Francis 1967), as reported in the MRC psycholinguistic database (Coltheart 1981). The adequacy of this matching was confirmed by a non-significant main effect of word type (cue/target) in an ANOVA, that included word frequency as the dependent variable, *F* (1, 158) = 1.63, *p* = .204, *η*_*p*_^2^ = .01.

Before participants completed the task, the entire procedure was explained to them, and they completed a practice of the task (consisting of five word pairs that did not overlap with the test stimuli) before beginning the experimental trials. As such, before studying the word pairs, participants were aware that their memory for each word pair would be tested. The task was run on an LG desktop computer and lasted approximately 25 min.

##### Study Phase

Firstly participants completed the study phase of the task. During this phase, participants were presented with the 80 cue-target word pairs. Word pairs were presented to participants sequentially and they were asked to memorise each pair shown on the screen, and then to click the mouse whenever they were ready to see the next pair. Whenever the mouse was clicked the next word pair appeared on the screen. As such, participants could take as long as they wanted to learn each pair. Word pairs were presented to all participants during the study phase in the same fixed, randomised order.

##### JOL Phase

After the study phase, there was a 5-min break. Participants then completed the JOL phase. During the JOL phase participants were individually presented, in a fixed random order (different to the order presented during the study phase), with the cue words alone. For example, if participants were presented with the cue-target word pair, *“bear*-*bridge”* during the study phase, during the JOL phase, they were presented with the cue word *“bear*-*?”* alone and asked to judge whether they thought they would be able to later recall the correct target word (*“bridge”*). For each cue word, participants were asked to make a JOL (either “Yes” or “No”) as to whether they would be able to recall the associated target word, when prompted with the cue word at a later point. Participants made their JOL response by pressing the “Y” key on the keyboard if they thought they would correctly remember the missing target word, and the “N” key if they did not think they would know the missing word (no time limit was imposed).

##### Cued-Recall Phase

Immediately after the JOL phase, participants completed a cued-recall test. They were presented with the cue words again sequentially, in a random order (again different to the order presented during the study or JOL phase), and were asked to recall the missing target word. Participants typed out their response, and submitted it by pressing the “enter” key. Once a recall response was submitted, the next cue word appeared on the screen. There was no time limit on this part of the task.

### Scoring

#### Memory Performance

Participants’ cued-recall memory performance on the JOL task was calculated as the proportion of target words correctly recalled during the cued-recall stage. The vast majority of recall responses were unambiguously correct or incorrect. However, on a very small number of occasions there was some debate as to whether a recall response should be considered correct. On such occasions recall responses were only considered correct if participants had (a) recalled a plural of the target word (e.g., if the target word was “*tree”,* a recall response of “*trees”* was considered correct), or (b) had clearly made a typing error when entering their response (e.g., if the target word was “*tree”,* a recall response of “*treew”* was also considered correct). Recall responses that were semantically similar to the target word, but were not the correct target word, were considered incorrect (e.g., if the target word was “*flask”,* a recall response of “*thermos”* was considered wrong).

#### Metacognitive Performance

Gamma scores (Goodman and Kruskal 1954) were calculated to provide an index of overall JOL *accuracy*. This analysis is recommended by Nelson (1984) and is commonly used to analyse JOL tasks (e.g. Wojcik et al. 2014). Gamma scores are a non-parametric measure of association (between participants’ predictions and actual performance) and were calculated by comparing the number of correct predictions that each individual made with the number of incorrect predictions they made. To calculate gamma scores, the formula *G* = (*ad* − *bc*)/(*ad* + *bc*) was used, with (a) representing the number of *correct* “Yes” predictions an individual made, (b) representing the number of *incorrect* “Yes” predictions, (c) representing the number of *incorrect* “No” predictions, and (d) representing the number of *correct* “No” predictions. Gamma scores range between + 1 and -1, where a score of 0 indicates chance-level accuracy, a large positive value indicates a good degree of accuracy, and a large negative value indicates less than chance-level performance on the task. However, when calculating gamma scores, the score cannot be calculated when two or more of the prediction rates (a, b, c, or d) are equal to 0. As such, the raw data were adjusted by adding 0.5 onto each prediction frequency and dividing by the overall number of JOL judgements made (*N*) plus 1 (*N* + 1). This correction is recommended by Snodgrass and Corwin (1988) and is routinely used when calculating gamma scores on metacognitive tasks (e.g., Bastin et al. 2012; Wojcik et al. 2013).

In addition to gamma scores, the proportion of errors made by participants in each group was calculated for two different *types of error in JOL predictions*. The proportion of *under*-*confident errors* participants made was calculated as the number of incorrect “No” predictions, in which they failed to predict their subsequently successful recall of a target word, divided by the total number of judgments made. The proportion of *over*-*confident errors* participants made was calculated as the number of incorrect “Yes” predictions made, in which they inaccurately predicted that they would recall a word that they subsequently failed to remember, divided by the total number of judgments made.

## Experiment 1: Results

### Judgment of Learning (JOL) Task

#### Memory Performance

The group difference in cued-recall memory performance was examined using an independent-samples *t* test (see Table 2 for descriptive and inferential statistics). This indicated that individuals in the ASD group recalled significantly fewer target words than comparison participants during the JOL task.

**Table 2:** Means (*SD*s) and inferential statistics for group differences in performance on the judgment of learning task used in Experiment 1

	Group		*t*	*p*	Cohen’s *d*
	ASD (*n* = 18)	Neurotypical (*n* = 18)			
Proportion of targets recalled	.30 (.26)	.49 (.25)	2.28	.029	0.74
Gamma score^a^	.76 (.13)	.71 (.14)	1.06	.295	0.37
Proportion of over-confident judgments	.09 (.06)	.11 (.07)	1.18	.247	0.31
Proportion of under-confident judgments	.03 (.05)	.03 (.03)	0.05	.958	0.00

^*a*^Gamma scores index metacognitive monitoring accuracy

#### Metacognitive performance

Group differences in metacognitive monitoring accuracy were also examined (see Table 2 for descriptive and inferential statistics). An independent-samples *t* test indicated that there was no significant difference in gamma score between the ASD and neurotypical groups. Thus, not in keeping with predictions, participants with ASD were not significantly poorer at predicting their own memory performance than were neurotypical participants, on the JOL task. One-sample *t* tests indicated that gamma scores were significantly above chance (i.e. significantly greater than 0) in both diagnostic groups, all *t*s > 21.16, all *p*s < .001.

An additional analysis was also carried out to investigate whether the significant group difference in cued-recall of target words confounded performance at the meta-level of the task (i.e., JOL accuracy). For the purpose of this analysis, two participants from each group were excluded to create ASD and neurotypical groups that were matched closely for recall ability, *t* (30) = 1.31, *p* = .200, *d* = 0.47. These sub-groups also remained matched for age, VIQ, PIQ, and FSIQ (all *p*s > .52, all *d*s < 0.23). An independent-samples *t* test indicated that even when groups were equated closely for recall ability, JOL gamma scores were still not significantly different in the ASD group (*M* = .75, *SD* = .13) than in the neurotypical group (*M* = .70, *SD* = .15), *t* (30) = 1.15, *p* = .261, *d* = 0.36.

Group differences in the specific type of errors participants made on the JOL task were also examined. Independent-samples *t* tests indicated no between-group differences in the proportion of under- or over-confident JOL errors (see Table 2 for statistics). One-sample *t* tests also indicated that the proportion of under- or over-confident JOL errors participants made was significantly above chance (i.e. significantly greater than 0), in both diagnostic groups, all *t*s > 2.47, all *p*s < .024.

### Relation Between JOL Accuracy and AQ Scores

In order to investigate the relation between metacognitive JOL accuracy and ASD/ASD-like traits, correlation analyses were performed among each diagnostic group. Among neither group was AQ total score significantly associated with JOL accuracy, all *r*s ≤ .33, all *p*s ≥ .183.

## Experiment 1: Discussion

The results of Experiment 1 suggest that JOL accuracy is undiminished among adults with ASD, contrary to expectations. However, it is important to consider alternative explanations before concluding that monitoring of own learning is intact in ASD. One possible explanation for the results of Experiment 1, and indeed Wojcik et al.’s (2014) findings, is that individuals with ASD used an atypical strategy to complete the task that required only very limited metacognitive monitoring. In other words, it might be possible that participants with ASD *performed* well on the delayed JOL task, despite diminished underlying metacognitive monitoring *competence*. Both this study and Wojcik et al. employed a standard JOL procedure in which participants were asked to make so-called “cue-alone” judgements (Dunlosky and Nelson 1992). During the JOL phase, participants were presented only with the cue word and were asked the judge the likelihood that they would later recall the corresponding target word. It may be that during a cue-alone JOL task, when presented with the cue word (e.g., “bear-?”) and asked to make a JOL about whether you will remember what the missing target word is at a later point, individuals adopt the strategy of simply answering “yes” if, *at the point they make the JOL*, they can remember the target word, and “no” if they cannot (in other words, they are not making a prediction as such). In this case, relatively accurate JOLs could be made on a cue-alone JOL task, simply by judging whether one can bring to mind the target word *at the time a JOL is made* (a strategy that involves no metacognitive monitoring processes). In other words, rather than monitoring the extent to which a target item has been successfully encoded and stored for *later* retrieval, participants could merely monitor whether or not the target item could be brought to mind currently. Importantly, this could also explain Wojcik et al.’s (2014) reported failure to find diagnostic group differences in JOL accuracy.

Additionally, whilst Experiment 1 suggested JOL accuracy is intact in adults with ASD, impairments in JOL accuracy may be developmental in nature and only apparent in children/adolescents with ASD. Experiment 2 addressed both these issues, and explored JOL accuracy in adolescents with ASD, using two JOL paradigms. An important variant to a standard JOL procedure involves participants making so-called “cue-target” rather than “cue-alone” judgements (Dunlosky and Nelson 1992). In this type of JOL task, individuals are asked to determine the future retrievability of the target when presented with *both* the cue and the target. During a cue-target JOL task, it is not possible to adopt a strategy of immediate self-testing of one’s current memory for a missing target item. In Experiment 2, adolescents with ASD and neurotypical adolescents completed both a cue-alone JOL task and a cue-target JOL task. It was predicted that adolescents with ASD would demonstrate impaired JOL accuracy on the cue-target JOL task, but not on the cue-alone JOL task.

## Experiment 2: Method

### Participants

Ethical approval for this study was obtained from the University of Kent Psychology Research Ethics Committee. Twenty-two adolescents with ASD (19 males, 3 females) and 21 neurotypical comparison adolescents (19 males, 2 females) took part in this experiment, after their parents had given written, informed consent. Participants in the ASD group had all received formal diagnoses of autistic disorder (*n* = 17) or Asperger’s disorder (*n* = 5). Parents of all children completed the Social Responsiveness Scale (SRS; Constantino et al. 2003), a parental report used to assess the severity of ASD features. T-scores of 60 and above are considered consistent with an ASD diagnosis (Constantino et al. 2003). In all but one case, participants with ASD scored above the ASD cut-off. The remaining participant, who scored 55 on the SRS, had a formal, verified diagnosis of an autism spectrum disorder, according to DSM-IV-TR criteria and was therefore included despite the slightly lower than expected SRS score.

Neurotypical participants were recruited from mainstream schools in the local area. The ASD and neurotypical groups were equated closely for VIQ, PIQ, FSIQ, and chronological age. Participant characteristics are presented in Table 3. All but four neurotypical adolescents scored below 60 on the SRS (the cut-off for ASD). One participant had a borderline score of exactly 60 and three others scored above the cut-off. None of the teachers or parents of these participants reported any history of ASD (or concern about a developmental disorder) in these four adolescents, and none of the participants had any diagnosis. It is unlikely, therefore, that these individuals had an undiagnosed ASD. However, to ensure that including these participants in the overall sample did not affect the results of the study, all experimental analyses in the paper were re-run excluding these participants (and excluding the one participant with ASD who scored below the SRS cut-off). We report the key experimental results *after* removing these participants in Footnote 1. The results were almost identical with and without these neurotypical participants included.

**Table 3:** Participant characteristics (means, standard deviations and inferential statistics) for the participants in Experiment 2

	Group		*t*	*p*	Cohen’s *d*
	ASD (*n* = 22)	Neurotypical (*n* = 21)			
Age (years)	13.70 (1.45)	13.21 (1.18)	1.21	.234	0.37
VIQ	100.68 (15.48)	98.76 (12.54)	0.45	.658	0.14
PIQ	101.41 (14.80)	102.86 (14.11)	0.33	.744	0.10
FSIQ	100.95 (14.06)	101.14 (13.68)	0.04	.965	0.01
SRS total score	83.14 (9.93)	47.29 (11.66)	10.87	<.001	3.31

*SRS* Social Responsiveness Scale (Constantino et al. 2003); *VIQ* verbal IQ, *PIQ* performance IQ, *FSIQ* full scale IQ

### Materials and Procedures

#### Judgment of Learning Tasks

**Fig. 2:**
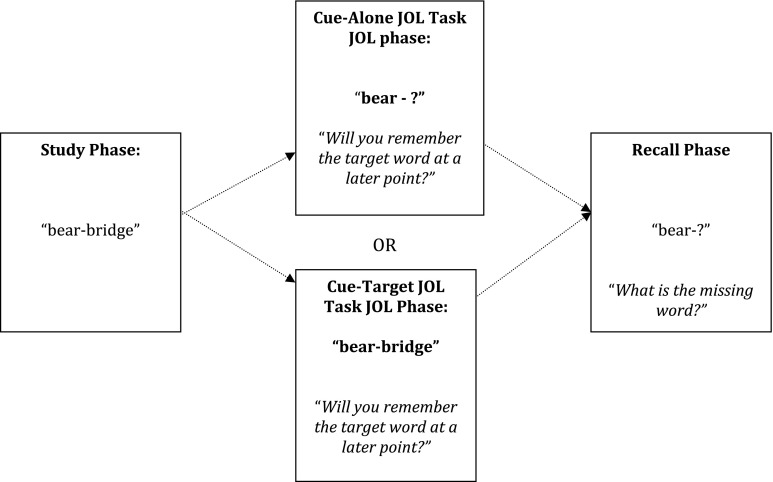
Graphical representations of the cue-alone and cue-target judgment of learning tasks used in Experiment 2

Two sets of 22 word pairs (44 words in total) were used as stimuli for the JOL tasks. Both sets were matched for mean syllable length and word frequency (Kucera and Francis 1967), as reported in the MCR psycholinguistic database (Coltheart 1981). To check that the words used in each set were adequately matched, a multivariate analysis of syllable length and word frequency across both sets was carried out. There was no main effect of set, as established by Wilks’ Lambda criterion, *F* (2, 85) = .152, *p* = .859, *η*_*p*_^2^ = .004. Participants were tested individually on both tasks during two separate testing sessions (please see Fig. 2 for a graphical representation of both JOL tasks). To avoid any order effects, the order participants completed each JOL task was counterbalanced. Before completing either task participants completed a practice block, consisting of five word pairs.

#### Cue-Alone JOL Task

The procedure employed during the cue-alone judgment of learning task used a delayed JOL design, consisting of a study phase, a JOL phase and a cued-recall test phase (and was similar to the task employed in Experiment 1). The task was run on a Sony VAIO laptop, and lasted approximately 15–20 min. During the study phase, participants were individually presented with 22 cue-target word pairs for 8 s each, in a fixed randomised order. Participants were told that their memory for each word pair would be tested at a later point, with the presentation of the *cue word alone.* After the learning phase participants then completed the JOL phase of the task, in which they were presented, in a fixed random order, with cue words alone (i.e., if participants learnt the cue-target pair “bear-bridge” then the JOL for this word pair was cued by the presentation of “bear-?”). The only previous study of JOL accuracy (Wojcik et al. 2014), alongside Experiment 1, asked participants to make dichotomous (Yes/No) JOL assessments. In both studies individuals with ASD did not demonstrate impairments in monitoring accuracy, relative to neurotypical individuals. However, it is possible that categorical judgements might not provide the variation necessary to observe group difference in JOL accuracy. As such, in this experiment participants were presented with each cue word individually for 5 s, and were asked to make a JOL on a scale of 1–5. It was explained to participants that a JOL of 1 indicated that they thought they would definitely *not* be able to remember the missing target word, and a JOL of 5 indicated they thought they would definitely be able to remember the missing target word. Immediately after the JOL phase, participants completed a cued-recall test. Participants were presented again with cue words alone, in a different, fixed random order, and were asked to recall the missing target word. Participants were not limited in the amount of time they had to recall the target word for a given cue word.

#### Cue-Target JOL Task

The cue-target JOL task followed the same procedure as the cue-alone JOL task. However, during the JOL phase instead of being presented with cue words alone, participants were presented with the *complete word pairs* again (i.e., if participants were presented with the cue-target pair “bear-bridge” at study, then the JOL for this word pair was cued by the presentation of “bear-bridge”). Apart from this difference, the procedure for each JOL task was exactly the same.

### Scoring

Participants’ basic memory performance was calculated as the proportion of target words correctly recalled during the cued-recall stage. Gamma correlations (Goodman and Kruskal 1954) were calculated to provide an index of overall JOL accuracy (please see above for a detailed description of gamma correlations). Gamma correlations were calculated based on all JOLs made.

## Experiment 2: Results

### Judgment of Learning (JOL) Tasks

#### Memory Performance

Table 4 shows the means and standard deviations for cued-recall performance on the cue-alone and cue-target JOL tasks. A mixed-model ANOVA was carried out on these data with Group (neurotypical/ASD) entered as the between-subjects variable, and JOL Test Type (cue-target/cue-alone) entered as the within-subject variable. There was a significant main effect of JOL Test Type on recall ability, reflecting the fact that, across participant groups, adolescents recalled significantly more target words in the cue-target JOL task (*M* = .49, *SD* = .21) than in the cue-alone JOL task, (*M* = .34, S*D* = .16), *F* (1, 41) = 31.14, *p* < .001, *η*_*p*_^2^ = .43. There was no significant main effect of Group, *F* (1, 41) = 0.01, *p* = .917, *η*_*p*_^2^ < .001, or Group × JOL Test Type interaction, *F* (1, 41) = 1.08, *p* = .305 *η*_*p*_^2^ = .03. Thus, adolescents with ASD demonstrated similar levels and patterns of recall to the neurotypical adolescents.

**Table 4:** Means (*SD*s) and inferential statistics for group differences in performance on both judgment of learning tasks used in Experiment 2

	Group		*t*	*p*	Cohen’s *d*
	ASD (*n* = 22)	Neurotypical (*n* = 21)			
Cue-alone recall performance	.32 (.11)	.36 (.21)	0.65	.517	0.24
Cue-target recall performance	.50 (.21)	.47 (.21)	0.33	.739	0.14
Cue-alone gamma score^*a*^	.89 (.12)	.92 (.12)	0.67	.505	0.25
Cue-target gamma score^*a*^	.45 (.41)	.53 (.45)	0.56	.582	0.19

^a^Gamma scores index metacognitive monitoring accuracy

#### Metacognitive performance

Table 4 shows the means and standard deviations for Gamma correlations on the cue-alone and cue-target JOL tasks among ASD and neurotypical participants. A mixed-model ANOVA was carried out on these data with Group (neurotypical/ASD) entered as the between-subjects variable, and JOL Test Type (cue-target/cue-alone) entered as the within-subject variable. There was a significant main effect of JOL Test Type on gamma scores, reflecting the fact that (as expected) adolescents in both groups had significantly lower gamma scores (i.e., lower accuracy) on the cue-target JOL task than on the cue-alone JOL task, *F* (1, 41) = 42.62, *p* < .001, *η*_*p*_^2^ = .51. The main effect of Group was non-significant, indicating that adolescents with ASD did not have lower gamma scores overall than neurotypical adolescents overall, *F* (1, 41) = 0.46, *p* = .504, *η*_*p*_^2^ = .01. Finally, contrary to predictions, there was also no significant Group x JOL Test Type interaction, *F* (1, 41) = 0.14, *p* = .706, *η*_*p*_^2^ < .01. Thus, there were no significant differences between ASD and neurotypical participants in terms of either levels or patterns of metacognitive performance on the two JOL tasks.^1^

### Relation Between JOL Accuracy and SRS Scores

In order to investigate the relation between metacognitive JOL accuracy and ASD/ASD-like traits, a series of correlation analyses was performed. Among participants with ASD, SRS score was not significantly associated with JOL accuracy on either cue-target or cue-alone JOL tasks, all *r*s ≤ .19, all *p*s ≥ .387. Likewise, among neurotypical participants, SRS score was not significantly associated with JOL accuracy on the cue-*target* task, *r* = .14, *p* = .534. However, SRS score was significantly negatively associated with JOL accuracy on the cue-*alone* task, *r* = −.51, *p* = .02.

## Discussion: Experiment 2

In Experiment 2, we sought to test the hypothesis that participants with ASD in Experiment 1 showed high JOL accuracy by employing atypical “non-metacognitive” strategies. It was predicted that it may be possible to show high accuracy on cue-alone JOL tasks by using atypical, non-metacognitive strategies, which should not be possible on cue-target JOL tasks. However, contrary to expectations, we found no evidence that adolescents with ASD show diminished JOL accuracy on either type of task. Rather, the diagnostic groups showed very similar levels and patterns of performance on both cue-alone and cue-target JOL tasks. Recall of target words was significantly better on the cue-target task than on the cue-alone task in both groups, which was entirely expected, given that adolescents were presented with the target words twice on the cue-target task, but only once during the cue-alone task. As such, the ASD group demonstrated typical cued-recall performance, compared to the neurotypical group. This is in keeping with findings within the literature that suggest, when cued-recall procedures are employed, individuals with ASD often exhibit relatively spared recall performance (for a discussion, see Bowler et al. 1997; Bowler et al. 2011). Across both groups, JOL accuracy was also significantly higher in the cue-alone task than in the cue-target task. This is in keeping with findings from the typically developing literature, that suggest individuals tend to demonstrate better metacognitive accuracy on cue-alone JOL tasks than cue-target JOL task (e.g., Dunlosky and Nelson 1992, 1997). However, the finding that there was no hint of *between*-group differences in JOL accuracy was unexpected.

## General Discussion

Until now, only one study has explored JOL accuracy in individuals with ASD (Wojcik et al. 2014). Based on their results, the authors of that study concluded that JOL accuracy is undiminished in ASD. However, there are several methodological concerns with Wojcik et al.’s study (outlined in the introduction) that we argue should lead to caution when interpreting results. Moreover, there was, in fact, some evidence of impaired *immediate* JOL accuracy among Wojcik et al.’s sample of participants with ASD, contrary to the authors’ interpretation. Given the uncertainty regarding the ability of individuals with ASD to monitor their own states of learning, we sought to investigate JOL accuracy among individuals with ASD (adults and adolescents) and closely matched comparison participants. Based on clear evidence that individuals with ASD have difficulties making metacognitive judgements other than JOL, we predicted that JOL accuracy would be diminished in ASD. However, we found no evidence of any kind that this was the case. Across both a cue-alone JOL tasks, and a cue-target JOL task adolescents and adults with ASD demonstrated entirely typical JOL accuracy.

In Experiment 2, among neurotypical participants, JOL accuracy on the cue-alone task was significantly associated with ASD/ASD-like traits (as measured by the SRS). In the neurotypical participants, higher levels of ASD-like traits were predictive of poorer JOL accuracy on the cue-alone task. This result is not in keeping with the equivalent correlation analysis carried out among adults in Experiment 1, which indicated that ASD-like traits (as measured by the AQ) were not related significantly to recall or JOL accuracy on a cue-alone task (in either group). It may be that there is a developmental difference in the extent to which JOL accuracy relates to ASD-like traits (i.e., that the association diminishes over time). Alternatively, the difference between studies might be explained by the fact that the AQ (a self-report measure) was used in Experiment 1, whereas the SRS (a parent report measure) was used in Experiment 2. Another alternative is that the significant correlation in Experiment 2 is merely a chance result. Certainly, we did not predict such an association and, as such, the result would not survive Bonferroni correction for multiple comparisons (with an adjusted alpha level of .0125). It is clear that further research is needed to disentangle these possibilities.

Before considering the explanations for and consequences of our findings, it is important to consider the issue of statistical power. Experiments 1 and 2 involved samples of *n* = 18 and *n* = 21/22 respectively. This is comparable with many published studies of ASD and, crucially, at least as large as (or larger than) many studies of metacognitive monitoring in ASD that report significant impairments in ASD. However, it is possible to question whether the null findings in the current study (or any study) are merely the result of insufficient power. While it is, of course, possible that between-group differences in JOL accuracy might become statistically significant in a larger sample of individuals with ASD, this would still not indicate a major difficulty with making judgements of learning in this disorder; In Experiment 1, participants with ASD demonstrated (non-significantly) *better* metacognitive accuracy than comparison participants (associated with a small effect size of *d* = 0.37) and, across both tasks in Experiment 2, participants with ASD showed only very slightly (and non-significantly) lower JOL accuracy (again associated with a small average effect size of *d* = 0.22). This consistency in our findings of only small, non-significant between-group differences in JOL accuracy (in two different samples across three different tasks) reduces the likelihood that the findings represent a Type II error. Nonetheless, we took two further steps to address this issue.

First, we have conducted a power analysis using G*Power3 (Faul et al. 2007) to establish the power of our study to detect the predicted between-group differences in JOL accuracy if they really existed (based on an assumed sample size of 18 participants per group in Experiment 1, and 21/22 participants per group in Experiment 2, and one-tailed tests). To do this, we needed to estimate an effect size for the predicted between-group differences.^2^ This estimation was not straightforward; As we have argued, some studies of metacognitive monitoring in ASD have potential confounds in the methods and/or data analyses employed. These confounds render the effect sizes reported for between-group differences in monitoring ability potentially unreliable. Nonetheless, if we pool the effect sizes from all eight studies of judgements of confidence and feelings of knowing judgements, we arrive at a Cohen’s *d* value of 0.68 for between-group differences in metacognitive ability (full details available from the authors upon request). Assuming this is reliable (although we think it is likely to be an underestimate), then our contrasts in Experiments 1 and 2 had between 64 and 71 % power to detect predicted between-group differences, which is somewhat below the 80 % power recommended by Cohen (1992).

Second, we calculated a Bayes factor for each of the between-group contrasts in JOL accuracy gamma, using an online Bayes calculator (Dienes 2008). Bayes factors provide a means of assessing the relative evidence for or against a given theory (e.g., Rouder et al. 2012). Bayes factors are particularly useful for interpreting null results, because they provide a means of establishing how relatively strong the evidence is in favour of one hypothesis (in this case, the null) over another hypothesis (see Dienes 2014). Jeffreys’ (1961) widely-used criteria for interpreting Bayes factors suggest that factors of >3 provide evidence for the alternative hypothesis, whereas values <1 provide evidence for the null hypothesis. Bayes factors between 1 and 3 provide inconsistent evidence for either hypothesis. The contrasts in Experiments 1 and 2 were associated with Bayes factors of between 0.04 and 0.34, which represents between “strong” and “moderate-to-anecdotal” evidence for the null hypothesis.

Previous findings suggest that individuals with ASD demonstrate diminished metacognitive accuracy on both feeling of knowing (e.g., Grainger et al. 2014; Wojcik et al. 2013) and judgment of confidence tasks (e.g., Grainger et al. 2016; Wilkinson et al. 2010). How can the current finding of undiminished JOL accuracy in the current study be reconciled with these findings? Currently there is ongoing debate within the literature as to whether individuals possess general metacognitive abilities, which remain stable across metacognitive tasks, or whether metacognitive ability is task specific (see e.g., Song et al. 2011; Kelemen et al. 2000). Interestingly several studies have found that an individual’s accuracy on one metacognitive task does not necessarily correlate with their accuracy on a different task (Souchay and Isingrini 2012; Souchay et al. 2004). Findings such as this have led some researchers to argue against the concept of a general metacognitive ability. If individuals do not hold a general metacognitive ability, it is possible that individuals with ASD are only impaired in some aspects of metacognition (feeling of knowing judgments/judgments of confidence) and not others (JOL).

Another explanation for the pattern of performance seen across studies among people with ASD comes from neuroimaging evidence. Chua et al. (2009) found that both feeling of knowing and judgment of confidence tasks appear to elicit activation in the right temporo-parietal junction (TPJ). However, the only two fMRI studies of JOL accuracy to date found no indication that TPJ was activated during JOL tasks (Do Lam et al. 2012; Kao et al. 2005). Given (a) substantial evidence that the right TPJ plays a pivotal role in representing others’ mental states (mindreading; see Perner et al. 2006; Samson et al. 2004; Saxe and Powell 2006); (b) that individuals with ASD show deficits on tasks that rely on TPJ (e.g., Castelli et al. 2002), and (c) that some theories predict impaired metacognition in ASD only to the extent that mindreading is impaired (e.g., Carruthers 2009), it is perhaps not inexplicable that judgment of confidence and feeling of knowing accuracy are selectively diminished (leaving JOL accuracy undiminished) among people with this disorder. Currently, there is not enough evidence to determine the best explanation for the pattern of results that appears in ASD (largely impaired feeling of knowing and judgment of confidence accuracy in ASD, alongside intact JOL accuracy). As such, it will be important that future research investigates this further, particularly studies that assess metacognitive accuracy across several tasks in the same individuals.

Whatever the explanation for the finding of undiminished JOL accuracy in the current study, the finding itself has important implications for educational practice. Judgements of learning are considered to have different functions for everyday learning and control of behaviour/cognition than feelings of knowing and judgements of confidence. For example, judgments of learning specifically are thought to be involved in guiding allocation of study time and self-paced study, which are determinants of learning success (e.g., Son and Metcalfe 2000). For example, individuals can use judgements of learning to (a) decide whether or not to study particular information (with sufficiently high judgments of learning leading to no study), and (b) decide on the order of priority that information should be studied in (see e.g., Metcalfe and Kornell 2005; Kornell and Metcalfe 2006). During efficient learning, people adaptively study material they believe they almost know first, then progressively more difficult material (i.e., studying items with high judgements of learning first, then material with low judgements of learning). Alternatively, learners might first choose to study items they judge as difficult (i.e., items given low judgements of learning; Dunlosky and Hertzog 1997; Thiede and Dunlosky 1999). Either way, efficient learning and decision-making rely to some extent on an individual being able to make relatively accurate judgments of learning. Indeed, JOL accuracy is a specific predictor of learning ability (e.g., Thiede 1999). Given that adults and adolescents with ASD appear as accurate as neurotypical individuals at JOL, it would be useful for teachers to encourage students with ASD to make explicit judgments of learning when trying to learn new material. It may be that adolescents with ASD do not make such judgements spontaneously, as neurotypical adolescents do, and so future research might usefully explore the effect on learning of asking versus not asking adolescents to make such judgements.

## Footnotes

1 As noted on in the “Participants” section of Experiment 2, four neurotypical participants scored above cut-off on the SRS and one participant with ASD scored below cut-off. If these participants are excluded, participant groups remain closely matched, with small effect sizes for between-group contrasts for all baseline characteristics (chronological age, VIQ, PIQ, and FSIQ; all *p*s ≥ .19, all *d*s ≤ 0.44). In terms of experimental JOL task performance, the ASD and neurotypical group also remained matched for cued-recall performance, on both the cue-target and cue-alone JOL tasks (all *p*s ≥ .37, all *d*s ≤ 0.29). Finally, when the mixed ANOVA reported in the “Metacognitive Performance” subsection of the “Experiment 2: Results” section was re-run with these participants excluded, the results were almost identical. There a significant main effect of JOL Test Type on gamma scores, *F* (1, 36) = 42.96, *p* < .001, *η*_*p*_^2^ = .54 (note: in the original analysis *η*_*p*_^2^ = .51). However, there was no significant main effect of Group, *F* (1, 36) = 0.20, *p* = .656, *η*_*p*_^2^ = .006 (note: in the original analysis *η*_*p*_^2^ = .01). Finally, there was no significant Group x JOL Test Type interaction, *F* (1, 36) = 0.17, *p* = .679, *η*_*p*_^2^ = .005 (note: in the original analysis *η*_*p*_^2^ < .01).

2 Note that a *post hoc* power calculation based on *observed* effect sizes in one’s data does not provide a means of judging the likelihood of whether a Type II error has been made (Baguley 2004).
